# Reaction to Koppad SN et al. Bilateral spermatocytic seminoma: an update on “synchronous” and “sequential” presentation of this rare variant of testicular tumour

**DOI:** 10.11604/pamj.2014.17.248.4118

**Published:** 2014-04-02

**Authors:** Piyush Kalakoti, Suman Sahu, Aarif Syed

**Affiliations:** 1 Lal Bahadur Shastri Hospital, New Delhi, DL 110091, India; 2 Chand Memorial Hospital, Patna, BR 800013, India

**Keywords:** Spermatocytic seminoma, synchronous, sequential, testicular tumour

## Abstract

We read with interest the report of Koppad and colleagues in the Pan African Medical Journal describing a case of bilateral synchronous presentation of spermatocytic seminoma in an elderly Indian male. While we appreciate their efforts in documenting this rare presentation, we disagree with the reported figures as outlined in the report and wish to draw attention of the authors as well as the readers of the journal to the gross inaccuracies in the reported statistics. We present our data, following a comprehensive literature review, to unveil the magnitude of bilateral presentation (synchronous and sequential) of this unique variant of testicular tumor as reported in medical literature to facilitate dissemination of precise information on the topic.

## Reaction

We read with interest the report of Koppad and colleagues [1] describing synchronous presentation of bilateral spermatocytic seminoma (SS) in an elderly male from rural India and appreciate their meticulous efforts in documenting this rare presentation. While the authors’ [1] assert bilateral synchronous SS has been reported previously, with two cases each from Japan and France and one from the United States, we strongly disagree with the reported statistics and wish to draw attention of the authors and the readership of the journal to the gross inaccuracies in the report. We herein present our data following a comprehensive literature review to unveil the magnitude of bilateral presentation (synchronous and sequential) of this unique variant of testicular tumor, and to help disseminate precise lineage of information on the topic.

An exhaustive literature search was conducted using electronic databases (MEDLINE^®^; EMBASE; CAB Abstracts; Current Contents and Google Scholar) for relevant, published, peer-reviewed articles up to mid February 2014. The search terms used were: seminoma; spermatocytic seminoma; bilateral spermatocytic seminoma with/without “synchronous” and “sequential”; testicular tumor and testicular cancer. Further, bibliographies of identified publications and articles citing them were also examined. Abstracts and reviews were as well included, without limitation on the language of publication. Inclusion of articles other than English language, where full texts and abstracts were unavailable for a possible translation, article selection relied mainly upon the title that had the search term “bilateral spermatocytic seminoma”. The resulting citations were exported to EndNote^®^ X7 and articles were screened independently by two authors (PK and SS) by reviewing their titles and abstracts. The following criteria were applied for screening: 1) testicular tumors relevant to SS; 2) limited to human subjects. Potential conflicts arising upon article selection were resolved by discussion and consensus. Data from identifiable papers was synthesized following which SS of bilateral origin was divided into 3 categories: synchronous [1–5] and sequential [6–8], and lastly where presentation could not be identified [9, 10] (Table 1). Even though bilateral, foreign language of the published literature and unavailability of full text or abstract for a possible review or translation limited our efforts to classify the latter into one of the two possible clinical presentations.

**Table 1:** Patient characteristics and clinical course of bilateral SS cases as reported in medical literature

Type of presentation	Age (years)	Tumor size (cm)	Duration of symptoms (years)	Tumor staging (TNM)	Management	Co-morbid condition/s	Follow up(years)	Outcome	Comment/s
**Synchronous presentation**									
Arvis G et al [2] †	NA	NA	NA	NA	NA	NA	NA	NA	Article in French
Arvis G et al [3] †	NA	NA	NA	NA	NA	NA	NA	NA	Article in French
Leocádio and Stein [4]	77	R: 5.4#	9	T1 N0 M0 (Stage Ia)	RO + post operative RT	Bilateral hydrocele; Erectile dysdunction	5	no recurrence	First case of synchronous bilateral SS reported in English literature
		L: 11.6#	9	T3 N0 M0 (Stage Ib)	RO + post operative RT				
Maruta S et al [5] ‡	56	NA	NA	T1 N0 M0(Stage I; pT1)	Bilateral high orchiectomy	-	0.42	no recurrence	Article in Japanese; details of the case extracted from the abstract
Koppad et al [1]	50	R: 5 x 4 x 3	1.5	NA	RO	Bilateral hydrocele	0.83	no recurrence	First case of synchronous bilateral SS from India
		L: 13 x 8 x 6	5	NA	RO				
**Sequential presentation**									
Bergner et al [6]	28	R: NA	1	NA	RO	-	NA	no recurrence	Unusual feature is the relatively young age of the patient
		L: NA	SS in the left testis 3 years after the initial diagnosis	NA	RO				
Chung et al [7]	68	Unclear about the laterality; one tumour measuring 4.5 and contra lateral 5.3	Unclear; SS in the contra lateral testis 9 years after the initial diagnosis	T1 N0 M0 (Stage 1)	Surveillance	-	10.4	no recurrence	-
				T1 N0 M0(Stage 1)					
Xu et al [8]	8	R: 7 x 4 x 4	0.17	NA	RO	-	1.5	no recurrence	-
		L: 7 x 4 x 3	2	NA	RO				
Could not be determined*									
Tmoyoshi and Kawamura [9]	NA	NA	NA	NA	NA	NA	NA	NA	Article in Japanese
Potet and Roland [10]	NA	NA	NA	NA	NA	NA	NA	NA	Article in French

SS spermatocytic seminoma; NA not available; R right side; L left side; RO radical orchiectomy; RT radiotherapy

# Measured along the long axis of the tumor

† Classified as synchronous as per data inferred from the title of the manuscript

‡ Classified as synchronous as per data inferred from the abstract of the manuscript available on PubMed

* Although bilateral, could not be classified into either synchronous or sequential due to unavailability of abstracts/full texts and with the information on the title of these manuscripts

Although spermatocytic seminoma, a distinctive testicular germ cell tumor, was first described by Pierre Masson[11] in 1946, there exists only five possible cases describing its synchronous presentation in the existing literature, including the one reported by Koppad et al [1] (Table 1). Ascertained by the title and year of publication (1969, 1970), it is estimated that first such case of synchronicity in presentation was reported by Arvis et al [2, 3]; however foreign language of publication (French), in addition to lack of full texts and abstract raises concern over its certainty. Interestingly, it could be argued that cases published by Tmoyoshi and Kawamur (1968, Japan) [9], and Potet and Roland (1968, France) [10] on bilateral SS could be describing a synchronous presentation; however a possible confirmation was complicated due to the above foresaid reasons. In contrast, the case of SS by Maruta et al [5] in a 56 year old Japanese male was ascertained to be of synchronous presentation from the data extracted from abstract, and subsequent translation of full text into English language. Perhaps, Leocádio and Stein [4] unarguably described the first case of synchronous SS reported in English literature (Table 1).

With only handful of reports documenting bilateral presentation of this unique variant of testicular tumor, we perceive that incidence of such involvement could be much higher than what is reported since its first description almost 7 decades ago. Keeping in view of a possible malignant transformation and the risk it poses, prospective studies looking at unraveling the unknown pathogenesis of these tumors should also note for its bilateral affliction, if at all, for a better understanding of its presentation and also to uncover more epidemiological data on the same.
